# Superoxide dismutase 6 is required during metamorphosis for the development of properly movable legs in *Tribolium castaneum*

**DOI:** 10.1038/s41598-022-10166-3

**Published:** 2022-04-27

**Authors:** Maaya Nishiko, Takuma Sakamoto, Seulgi Mun, Mi Young Noh, Yasuyuki Arakane, Michael R. Kanost, Katsuhiko Arai, Hiroko Tabunoki

**Affiliations:** 1 Department of United Graduate School of Agricultural Science, Tokyo University of Agriculture and Technology, 3-5-8 Saiwai-cho, Fuchu, Tokyo, 183-8509 Japan; 2 Institute of Global Innovation Research, Tokyo University of Agriculture and Technology, 3-5-8 Saiwai-cho, Fuchu, Tokyo, 183-8509 Japan; 3 Department of Science of Biological Production, Graduate School of Agriculture, Tokyo University of Agriculture and Technology, 3-5-8 Saiwai-cho, Fuchu, Tokyo, 183-8509 Japan; 4 Department of Applied Biology, Chonnam National University, Gwangju, 61186 South Korea; 5 Department of Forest Resources, AgriBio Institute of Climate Change Management, Chonnam National University, Gwangju, 61186 South Korea; 6 Department of Biochemistry and Molecular Biophysics, Kansas State University, 141Chalmers Hall, Manhattan, KS 66506-3702 USA; 7 Department of Tissue Physiology, Tokyo University of Agriculture and Technology, Fuchu, Tokyo, 183-8509 Japan; 8 Cooperative Major in Advanced Health Science, Graduate School of Bio-Applications and System Engineering, Tokyo University of Agriculture and Technology, Fuchu, Tokyo, 183-8509 Japan

**Keywords:** Entomology, Biochemistry, RNAi

## Abstract

The body form of holometabolous insects dramatically transforms from larval to adult stages during metamorphosis that occurs in the pupal stage. The larval disorganization and then new adult tissues are built up at this time. In motoneuron, larval neuronal cells degenerate, and new adult neurons are remodeled. Finally, adult neurons reconnect to new adult muscles. However, the factors that control metamorphosis have not yet been fully elucidated. Here, we show that an antioxidant enzyme, *Tribolium castaneum* superoxide dismutase 6 (TcSOD6), is secreted into the haemolymph and is required for proper movable legs during metamorphosis. *TcSOD6* has a unique domain architecture and is mainly expressed in the pupal stage. The depletion of *TcSOD6* expression in the pupa inhibits normal axon development and results in adults that display dysfunctional leg motions, suggesting that SOD6 expression is required for the development of properly movable legs. Therefore, we speculate that *TcSOD6* might participate in some of the processes for larval neurons to be remodelled to new adult functions in the legs during metamorphosis, providing new insight into the evolution of SOD functions.

## Introduction

Oxidative stress is caused by the generation of reactive oxygen species (ROS), which are toxic to organisms because of causing oxidative damage to proteins, lipid oxidation, and DNA damage^1,2^. However, organisms have innate systems for detoxifying ROS and controlling their levels^1,2^. Superoxide dismutase (SOD) scavenges superoxide anions (O_2_^−^) and converts them into hydrogen peroxide^3^. SOD proteins are metalloenzymes that are widely distributed in prokaryotes and eukaryotes; they are classified as copper/zinc SOD (Cu/Zn SOD; SOD1) and manganese SOD (Mn SOD; SOD2)^4^. Insect SODs were first identified in *Drosophila melanogaster*^5^. Soluble cytoplasmic SOD1 is a copper- and zinc-containing enzyme^5^. SOD2 is a mitochondrial matrix enzyme that scavenges oxygen radicals produced by the oxidation–reduction and electron transport reactions that occur in mitochondria^5^. SOD3 is an extracellular copper- and zinc-containing enzyme present in the haemolymph, intercellular fluid, and molting fluid^5^. In addition, copper chaperone superoxide dismutase (CCS) is present in *D. melanogaster*^5^.

Our group previously reported seven types of SODs in the lepidopteran insect *Bombyx mori*^6^. The analysis of amino acid sequences revealed that *BmSOD1*, *BmSOD3*, *BmSOD4*, *BmSOD5*, *BmSOD6*, and *BmCCS* likely bind copper and zinc ions, while *BmSOD2* binds manganese ions. Domain architecture suggests that *BmSOD1* and *BmCCS* are mainly localized in the cytosol, *BmSOD2* is localized in mitochondria, and *BmSOD3*, *BmSOD4*, *BmSOD5*, and *BmSOD6* are secreted into the extracellular space^6^. These BmSOD genes play a role in controlling the levels of ROS in different tissues during development^6^. *Bombyx mori* is a holometabolous insect characterized by extensive structural remodelling during the pupal stage in a process known as metamorphosis^7^. Our previous work showed that the BmSOD1 and BmSOD2 proteins are downregulated at pupation^8^ and that pupation is slightly disrupted in *B. mori* larvae by injection with an SOD mimic^9^. In addition, ROS production significantly increases before pupation in the fat body of *B. mori*^9^, and ROS stimulate the expression of programmed cell death-related genes^9^. Hence, the generation of ROS to induce programmed cell death is part of the progression of metamorphosis.

BmSOD6 has a unique domain architecture consisting of a secretion signal peptide, three copper/zinc SOD domains, and a transmembrane segment. BmSOD6 mRNA expression is increased at the pupal stage^6^. SOD6 orthologues have been identified in the genomes of several insect species, including *B. mori*, *Tribolium castaneum*, *Teleogryllus occipitalis*, *D. melanogaster*, *Anopheles gambiae*, and *Apis mellifera,* but not in vertebrates [EnsemblMetazoa; http://metazoa.ensembl.org/index.html]. We are interested in the function of SOD6 in insects, which has not previously been investigated.

**Figure 1:**
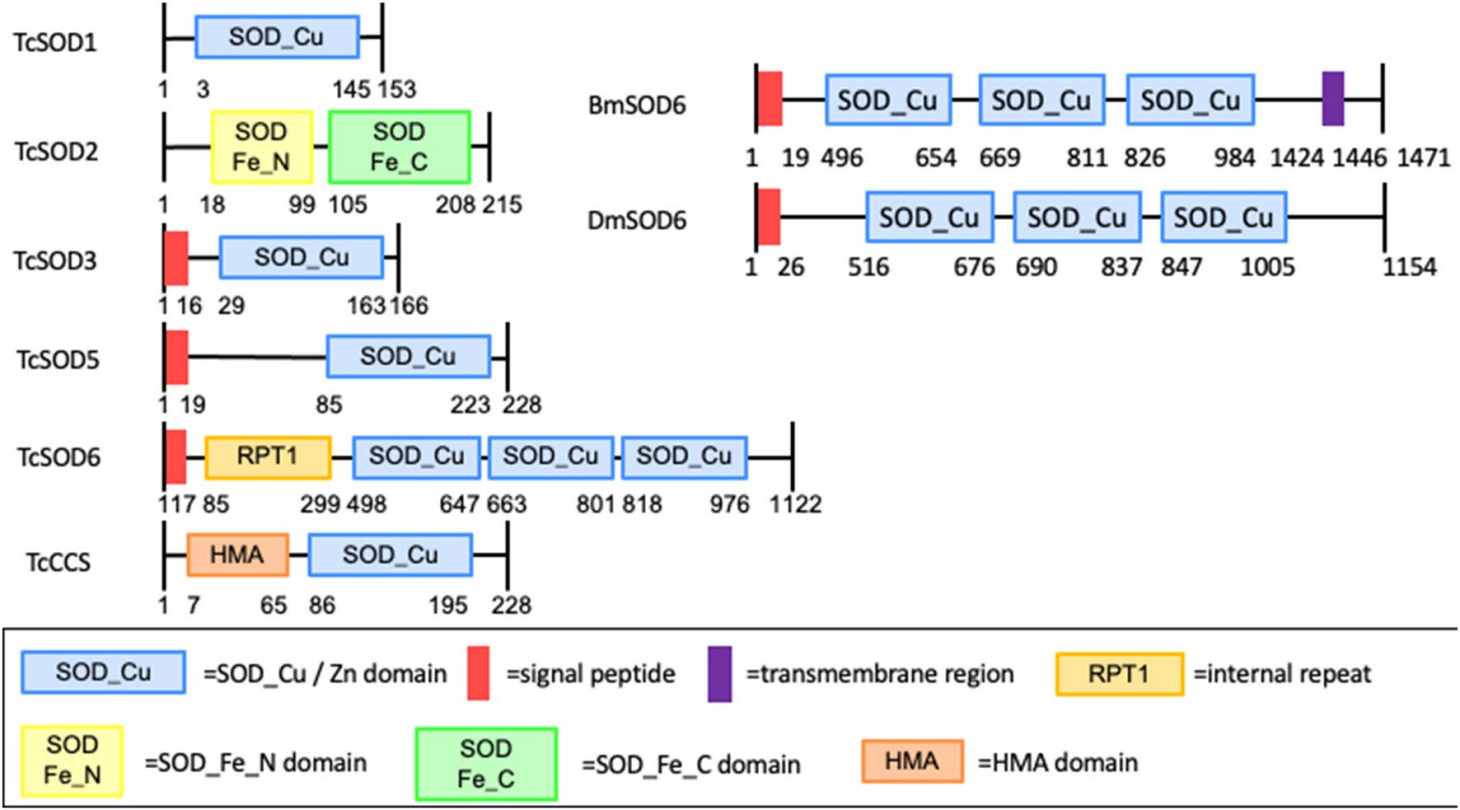
Domain organization of insect superoxide dismutase. Upper digits show the amino acid position. Sod_Cu and Sod_Fe are the distinguishing SOD domains. Blue box, SOD_Cu/Zn domain; red box, signal peptide; purple box, transmembrane segment; yellow–orange box, repeat (RPT) domain; yellow box, SOD_Fe_N domain; green box, SOD_Fe_C domain; and red–orange box, HMA domain. *T. castaneum* superoxide dismutase (TcSOD)-1, 2, 3, 5, and 6, *T. castaneum* copper chaperone for superoxide dismutase I (TcCCS), *B. mori SOD6* and *D. melanogaster SOD6*.

The red flour beetle (*T. castaneum*) belongs to the order Coleoptera. The genome of this species is well characterized, and systemic RNA interference (RNAi) works very efficiently in *T. castaneum*^10–13^. There are six SOD genes in the *T. castaneum* genome database, which are classified based on similarity to *B. mori* SODs as *TcSOD1*, *2*, *3*, *5*, and *6*, and *TcCCS* (Fig. 1). In this study, we investigated the functions of the *T. castaneum* SOD6 gene (*TcSOD6*) by RNA interference, transcriptome analysis, and histochemistry, and we identified a novel physiological function in *TcSOD6*-knockdown beetles, which showed dysfunctional leg movement as adults.

## Results

### Identification and characterization of the TcSOD6 sequence

We obtained *TcSOD6* and other *TcSOD* sequences from Beetlebase [now available at NCBI, https://www.ncbi.nlm.nih.gov/genome/216?genome_assembly_id=271841] (Supplementary Table 1) and cloned the *TcSOD6* cDNA to confirm its amino acid sequence. The cloned nucleotide sequence of TcSOD6 was submitted to the DNA Data Bank of Japan/European Nucleotide (DDBJ/ENA); Accession no. LC430326. The deduced open reading frame (ORF) of *TcSOD6* was 3366 bp long, encoding a protein of 1122 amino acid residues with a predicted molecular mass of 124,909 Da and a putative isoelectric point of 6.96 after the removal of the 17-residue secretion signal sequence.

A protein motif search revealed that TcSOD6 amino acid sequences contain three copper/zinc superoxide dismutase domains (Sod_Cu/Zn, Pfam; PF00080) at positions P498-I647, N663-R801, and H818-I976 and a repeat (RPT) domain at E85-I299 (Fig. 1). We found that the *TcSOD6* sequence (LC430326) contained several amino acid substitutions compared with the *TcSOD6* sequence predicted from the gene model (TC011770) (Supplementary Fig. 1, black box).

**Figure 2:**
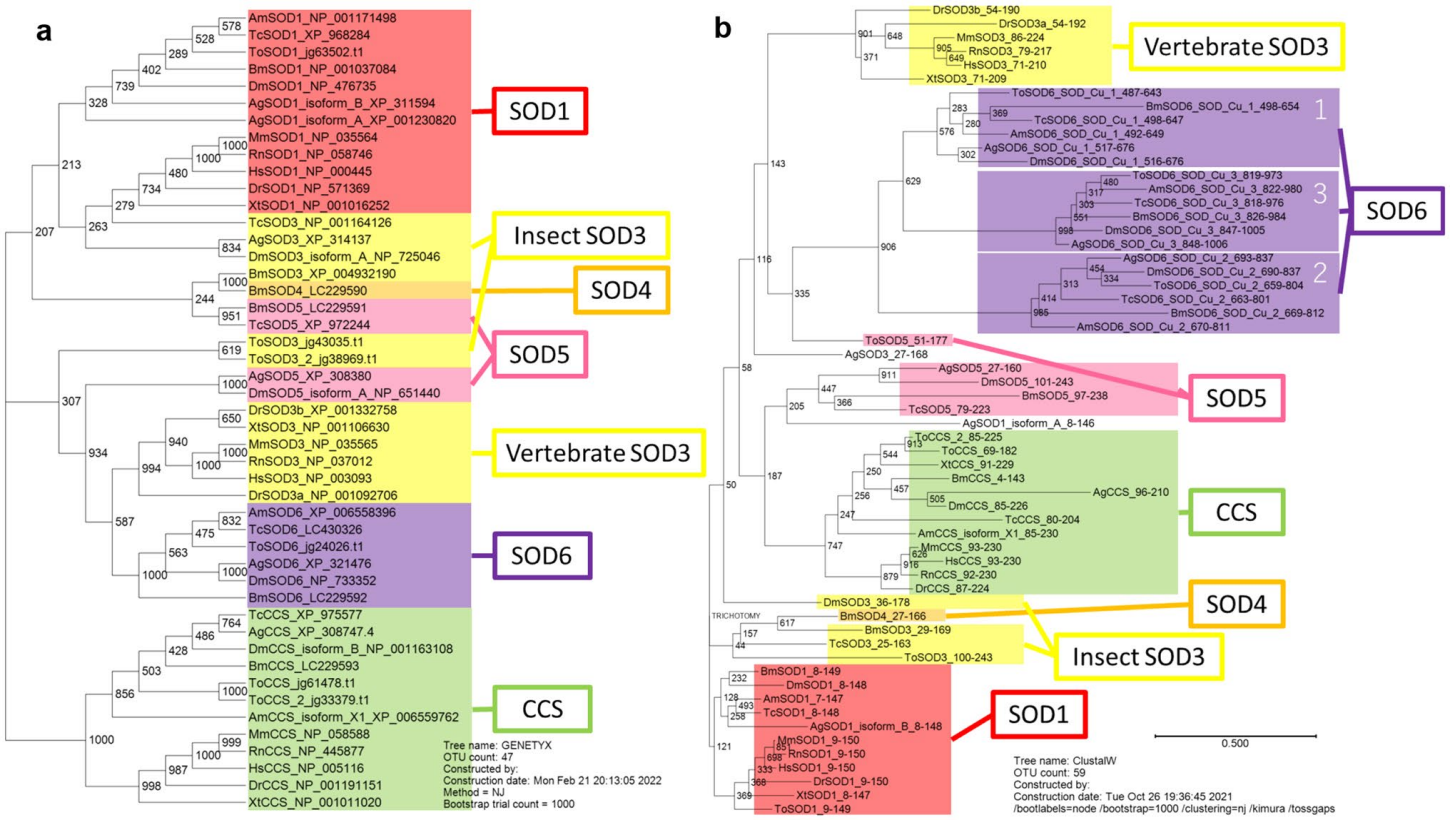
Phylogenetic analysis of superoxide dismutases. (**a**) A phylogenetic tree of six types of *Tribolium castaneum* superoxide dismutases (SOD) and SOD proteins of other species. (**b**) A phylogenetic tree of SOD_Cu/Zn domains in *T. castaneum* and other species. The amino acid sequences of the SODs used in this study are presented in Supplementary Tables 1 and 2. The SOD gene name of each animal is presented as the acronym of its scientific name plus SOD (e.g., *Tribolium castaneum* superoxide dismutase [TcSOD]).

In a phylogenetic tree containing the amino acid sequences of *TcSOD6* and the copper/zinc *SOD*s of some other insect species and selected vertebrates (Supplementary Table 2), the five identified *TcSODs* and the *SOD*s of other species were distributed among seven clusters (Fig. 2a). The phylogenetic tree revealed that *TcSOD6* was grouped into a cluster including only insect *SOD6*s (Fig. 2a). The cluster including *SOD6* that we identified was more closely related to vertebrate *SOD3* than to vertebrate *SOD1* and insect *SOD3* (Fig. 2a). To investigate evolutionary distances, we also examined the amino acid sequences of the SOD_Cu/Zn domains of each SOD by phylogenetic analysis. The SOD_Cu/Zn domains of SOD6 from each insect species formed a separate clade, suggesting that the three domains arose by duplication after the origination SOD6 during insect evolution (Fig. 2b). TcSOD6 was similar to and appeared to be an orthologue of *B. mori* SOD6 (Accession ID; LC229592, 87%), *D. melanogaster* SOD6 (Gene ID; 43586, 88%), *Anopheles gambiae* SOD6 (Gene ID; 1281551, 89%), and *Apis mellifera* SOD6 (Gene ID; 413369, 88%).

Next, we examined predicted functional amino acid residues in the three SOD_Cu/Zn domains of TcSOD6 by comparison with SOD_Cu/Zn domains from BmSOD6, DmSOD6, *Homo sapiens* SOD1 (P00441), and SOD3 (P08294) (Supplementary Fig. 2). The cysteines forming the disulfide bond^14,15^, the histidine required for binding to hydroxyperoxide, and the amino acid residues responsible for metal binding^14,15^ were conserved in the second SOD_Cu/Zn domains in TcSOD6. These amino acid residues play a role in the enzymatic function of SOD^16^. Most functional amino acid residues were conserved in each SOD_Cu/Zn domain of TcSOD6 (Supplementary Fig. 2a–c), but the zinc-binding ligand amino acids were conserved at only one position in the second SOD_Cu/Zn domain (Supplementary Fig. 2b).

### Developmental expression of TcSOD6 mRNA

**Figure 3:**
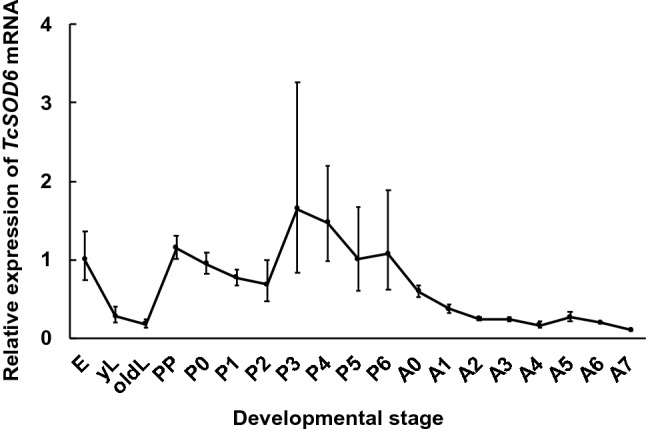
*TcSOD6* mRNA expression during development. mRNA expression of *TcSOD6* during development. E; embryo (n = 30), yL; young larva (n = 3), oldL; old larva (n = 3), PP; pharate pupa (n = 3), P0–P6; day 0–6 of the pupal stage (n = 3, respectively), A0–A7; day 0–7 of the adult stage (n = 3, respectively). For these samples, mRNA expression levels in the whole body are presented as RQ values, which represent the relative expression levels calculated using embryo samples as 1. Error bars indicate standard deviations from three individual experiments. *TcRpS6* was used as an endogenous control.

The expression of *TcSOD6* mRNA was investigated from the egg to adult-day 7 developmental stages by RT–qPCR. After the egg stage, *TcSOD6* mRNA expression decreased during larval development, increased during the pupal stage when the insect undergoes metamorphosis, and gradually decreased after molting to the adult stage (Fig. 3). Overall, *TcSOD6* mRNA expression was highest in the pupal stage.

### Verification of TcSOD6 knockdown

**Figure 4:**
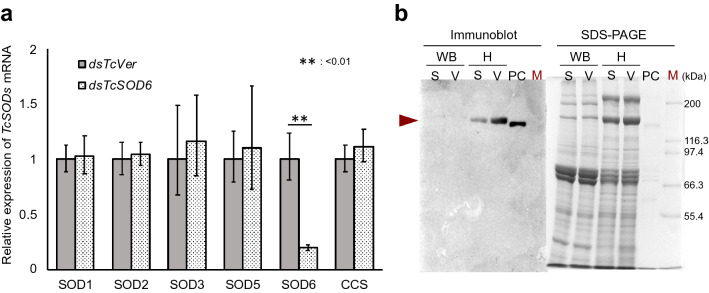
Verification of the expression of *TcSOD6* mRNA and protein after the knockdown of *TcSOD6*. (**a**) Verification of the mRNA expression levels of *TcSODs* by qRT–PCR in *TcSOD6* knockdown-*T. castaneum*. Whole bodies subjected to *TcSOD6-* and *TcVer-*knockdown (n = 3, respectively) were used for RT–qPCR. RQ values of *TcVer*- and *TcSOD6*-knockdown pupae. The bar charts represent the mean RQ value. The error bars represent the relative minimum / maximum expression levels relative to the mean RQ value. (**b**) Immunoblot analysis of TcSOD6 proteins. Proteins in pupal whole body lysates (WB) and haemolymph (H) were separated by SDS–PAGE and then analysed by immunoblotting using an antibody against the TcSOD6 protein. The TcSOD6 protein band was detected at ~ 130 kDa, as indicated by the arrowhead. Lanes labelled (M) include molecular weight standard proteins. (S) indicates ds*TcSOD6*; (V) indicates ds*TcVer*; (PC) indicates the recombinant TcSOD6 protein (0.55 μg/lane) used as a positive control. Full-length blots/gels are presented in Supplementary Fig. 4.

We carried out *TcSOD6* dsRNA injection to deplete the *TcSOD6* transcript to investigate its function. The *TcSOD6* dsRNA knockdown efficiency was examined 5 days after the injection of dsRNA into prepupae by RT–qPCR. As a negative control, dsRNA for *T. castaneum* tryptophan oxygenase (*TcVer*), required for normal eye pigmentation, was injected. This dsRNA treatment led to a significant decrease in the transcript of *TcSOD6* without any effect on those of other *TcSODs* (Fig. 4a). We also investigated the expression of *TcSOD6* at the protein level via immunoblot analysis of pupal haemolymph and whole-body protein lysates. The TcSOD6 protein was detected in the haemolymph as a band at the expected size of ~ 130 kDa (Fig. 4b). The antiserum for the TcSOD6 protein specifically recognized the TcSOD6 protein, and the intensity of the TcSOD6 protein band was decreased after *TcSOD6* knockdown.

### TcSOD6 knockdown in the prepupa affects leg movement in adults

**Figure 5:**
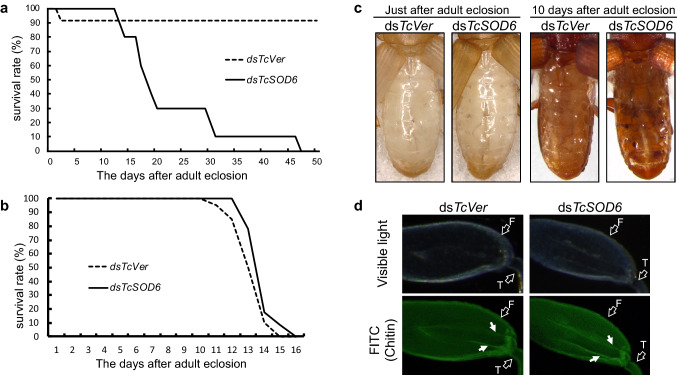
Phenotype of *TcSOD6*-knockdown beetles. (**a**) Effect of *TcSOD6*-knockdown on the adult lifespan of *T. castaneum*. Prepupae were treated with *TcSOD6* or *TcVer* dsRNA. The number of live beetles was counted from adult day 0 for a period of 16 or 50 days. (**b**) without feeding. The solid line indicates *TcSOD6*-knockdown adults, and the dashed line indicates *TcVer*-knockdown adults. (**c**) The phenotype of the abdomen in the *TcSOD6*- or *TcVer*-knockdown group immediately after eclosion (left two panels) and at 10 days after adult eclosion (right two panels). (**d**) Observation of the tendon structure of *TcVer*- and *TcSOD6*-knockdown adult hind legs by histochemical analysis. The tendon was stained with FITC-CBD (to detect chitin) and observed under a fluorescence microscope. White arrows indicate tendons. The open arrows indicate the parts of the hind leg; F, femur; T, tibia.

Adults that had been injected with dsRNA in the pharate pupae were examined to assess the phenotype of the *TcSOD6-*knockdown insects. *TcSOD6*-knockdown beetles had a much shorter lifespan, with only 10% of the beetles surviving at 30 days (Fig. 5a) versus 90% survival in controls. When the two knockdown groups were starved, the survival rate did not differ between them (Fig. 5b). Abdominal morphology in each knockdown group was assessed immediately after adult eclosion and at 10 days after adult eclosion. The size of the visible fat body in the abdomen was decreased at 10 days after adult eclosion in *TcSOD6*-knockdown beetles compared with *TcVer*-knockdown control beetles (Fig. 5c).

*TcSOD6*-knockdown adults showed slower, more rigid leg movements than *TcVer*-knockdown adults (Supplementary Videos 1 and 2). The *TcSOD6*-knockdown adults were unable to walk normally due to dysfunctional leg movement and were not able to turn over by themselves after being placed on their back. The legs of the *TcSOD6*-knockdown adults seemed to lack coordinated control. The *TcSOD6*-knockdown phenotype appeared in all beetles injected with *TcSOD6* dsRNA (n = 12). Altered leg movement was mainly observed in the T2 and T3 legs. All beetles with *TcSOD6*-knockdown displayed abnormal movement of the T2 and T3 legs (n = 12). In addition, five beetles also exhibited abnormal movement of the T1 leg (Supplementary Table 3). However, when *TcSOD6* dsRNA was injected immediately after adult eclosion (day 0 adult, n = 5), leg movement was not affected by treatment with *TcSOD6* dsRNA (Supplementary Videos 3 and 4 and Supplementary Table 4). Thus, we concluded that impaired leg movements appeared only when *TcSOD6*-knockdown occurred during the pupal stage. The structure and morphology of the tendons supporting the leg movement of *T. castaneum* were assessed by histological analysis. As shown in Fig. 5d (arrows), there was no apparent difference in the morphology of these structures between the *TcVer-* and TcSOD6-knockdown adults (Fig. 5d). These results suggested that movement disability may have decreased the ability of *TcSOD6*-knockdown adults to obtain a sufficient amount of food, resulting in a lack of stored lipids and a shortened lifespan.

### Analysis of the cause of leg movement impairment by TcSOD6 knockdown

**Figure 6:**
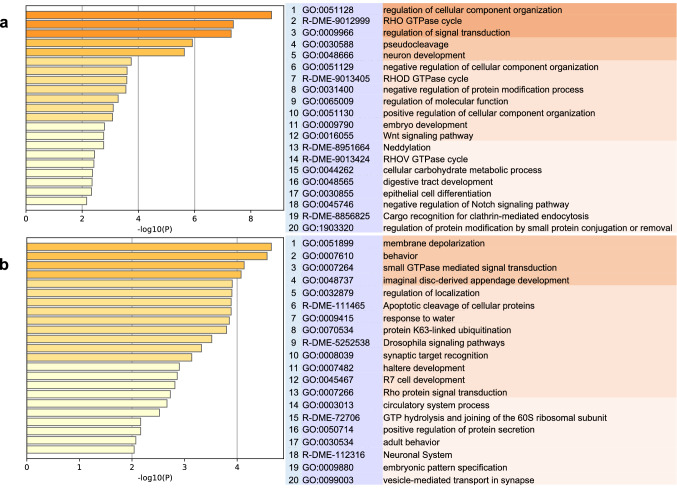
The gene transcript levels in the *TcSOD6*-knockdown groups were compared with those in the *TcVer*-knockdown groups by RNA-seq analysis. Transcripts with different expression levels between the *TcSOD6* dsRNA-injected group and the controls were used for gene enrichment analysis. A heatmap of enriched terms across the input transcript lists; differently coloured bars indicate *P* values. (**a**) Upregulated genes in the *TcSOD6* dsRNA-injected group (**b**). Downregulated genes in the *TcSOD6* dsRNA-injected group.

We further investigated whether *TcSOD6*-knockdown resulted in changes in the expression of other transcripts. RNA-Seq analysis was performed on three samples in the *TcVer*-knockdown groups (SRA accession numbers: DRR232570, DRR232571, and DRR232572) and three samples in the *TcSOD6*-knockdown groups (DRR232567, DRR232568, and DRR232569). These individuals were injected with each dsRNA within 24 h before pupation, and the total RNA samples were collected 4 days after pupation. The RNA-Seq data were mapped to the gene set retrieved from the NCBI database (Tcas5.2). In total, 37,895 transcripts were obtained from the RNA-Seq data, 317 of which were differentially expressed (false discovery rate < 0.05; Fig. 6a) between the *TcVer-* and *TcSOD6*-knockdown groups. Among the differentially expressed transcripts identified in the *TcSOD6*-knockdown group, 147 were upregulated and 170 were downregulated. For gene enrichment analysis with the Metascape gene annotation and analysis resource, the *T. castaneum* gene ID numbers were converted to *D. melanogaster* gene ID numbers using the tBLASTx algorithm to compare the six-frame translations of the nucleotide query sequence against the six-frame translations of a nucleotide sequence database. Among the 147 upregulated and 170 downregulated genes, 127 and 114 genes corresponded to *D. melanogaster* genes, respectively. The 20 Gene Ontology (GO) functional groups generated with Metascape from the upregulated transcripts of the *TcSOD6*-knockdown groups included the RHO GTPase cycle (R-DME-9012999) and neuron development (GO:0048666) categories (Fig. 6a). The downregulated transcripts of the *TcSOD6*-knockdown groups included the behaviour (GO:0007610), small GTPase-mediated signal transduction (GO:0007264), synaptic target recognition (GO:0008039), adult behaviour (GO:0030534), and the neuronal system (R-DME-112316) categories (Fig. 6b).

Several neuronal development-related genes were included in the differentially expressed transcripts. The top 100 transcripts with different counts between the *TcSOD6-* and *TcVer-*knockdown groups were identified. The upregulated transcripts included MSTRG.3637.7 (*p* = 0.0163); MSTRG.10482.1 (*p* = 0.1183); MSTRG.10408.88 (*p* = 0.0484); rna-XM_015982120.1 (*p* = 0.0009); MSTRG.2172.1 (*p* = 0.1348), corresponding to ankyrin 2, isoform N; Rho GTPase activating protein at 19D, isoform B; down syndrome cell adhesion molecule 1, isoform BP; Na channel protein 60E, isoform K; and Rho guanine nucleotide exchange factor 2, isoform H (Table 1). The downregulated transcripts included MSTRG.3723.4 (*p* = 0.0011); rna-XM_015982158.1 (*p* = 0.0436); MSTRG.1955.1 (*p* = 0.0025); rna-XM_008201549.2 (*p* = 0.0229); MSTRG.4493.10 (*p* = 0.0001), corresponded to sprint, isoform J; paralytic, isoform AN; myosin binding subunit, isoform P; sidestep, isoform D; and turtle, isoform I (Table 2).

**Table 1:** The count data for upregulated transcripts in the *TcSOD6*-knockdown groups.

Rank	Transcript_id	dsTcSOD6-1	dsTcSOD6-2	dsTcSOD6-3	dsTcver-1	dsTcver-2	dsTcver-3	NCBI IDs
1	MSTRG.81.4	2972	2912	3741	297	831	683	NP_001014708.1
2	rna-XM_008198392.2	505	507	487	355	306	515	NP_001027262.1
3	MSTRG.11025.2	4189	5733	0	0	0	0	NP_001097005.1
4	MSTRG.3637.7	325	703	387	13	0	0	NP_001097533.1
5	rna-XM_008199988.2	97	70	58	31	35	29	NP_001097544.1
6	rna-XM_008195996.2	300	660	8	4	3	0	NP_001137985.1
7	rna-XM_008199826.2	266	276	269	64	89	140	NP_001138108.1
8	MSTRG.10482.1	5895	1116	2904	313	769	500	NP_001162809.1
9	MSTRG.6412.12	7947	7434	6467	690	2270	707	NP_001245422.1
10	MSTRG.10408.88	2931	1421	973	50	135	142	NP_001246175.1
11	rna-XM_015985493.1	8	106	41	3	3	3	NP_001246242.1
12	rna-XM_008201901.2	427	492	189	53	128	85	NP_001246502.1
13	rna-XM_008201902.2	450	555	239	80	144	80	NP_001246502.1
14	rna-XM_015982120.1	761	513	632	0	0	0	NP_001246509.1
15	rna-XM_970845.4	15,428	12,224	5	0	0	0	NP_001247028.1
16	rna-XM_015979019.1	6	14	113	0	0	0	NP_001259109.1
17	rna-XM_015979235.1	8	27	20	0	0	0	NP_001259192.1
18	rna-XM_015983539.1	389	141	197	8	0	3	NP_001259643.1
19	rna-XM_008196835.2	2000	952	259	0	0	0	NP_001259764.1
20	rna-XM_008195910.2	146	59	97	0	0	0	NP_001260230.1
21	rna-XM_015978728.1	99	77	102	17	13	32	NP_001260441.1
22	MSTRG.10350.33	391	374	308	286	238	407	NP_001260528.1
23	rna-XM_966320.4	2780	2532	2103	569	889	1366	NP_001260795.1
24	rna-XM_015980327.1	291	127	68	44	32	49	NP_001260810.1
25	rna-XM_015979690.1	113	261	170	25	56	49	NP_001260850.1
26	MSTRG.5061.22	2364	2070	1782	2005	1381	2221	NP_001261051.1
27	rna-XM_015980072.1	9	509	105	0	0	0	NP_001261141.1
28	rna-XM_015982121.1	35	88	23	0	0	0	NP_001261172.1
29	MSTRG.3115.1	508	532	456	434	372	532	NP_001261204.1
30	MSTRG.3837.7	6347	5331	4940	3791	3257	4864	NP_001261284.1
31	MSTRG.7778.9	2092	2386	742	23	0	0	NP_001262460.1
32	rna-XM_008201788.2	459	408	314	279	255	278	NP_001262865.1
33	rna-XM_015981731.1	9	147	18	0	0	0	NP_001286447.1
34	MSTRG.2172.1	3660	2238	2694	1442	1190	2589	NP_001286515.1
35	MSTRG.5162.13	21,571	22,693	16,110	2844	2599	2791	NP_001287262.1
36	MSTRG.424.3	123	1436	2	0	0	0	NP_001287404.1
37	rna-XR_001575439.1	189	141	124	70	50	102	NP_001303505.1
38	rna-XM_015980478.1	46	64	76	0	0	0	NP_001334674.1
39	rna-XM_015980473.1	47	54	40	0	0	0	NP_001334674.1
40	rna-XM_015980474.1	46	51	40	0	0	0	NP_001334674.1

These count data indicate the number of sequence fragments that have been assigned to each gene. (n = 3, biological replication).

**Table 2:** The count data for downregulated transcripts in the *TcSOD6*-knockdown groups.

Rank	Transcript_id	dsTcSOD6-1	dsTcSOD6-2	dsTcSOD6-3	dsTcver-1	dsTcver-2	dsTcver-3	NNCBI IDs
1	MSTRG.10034.1	953	1049	841	1588	1332	2085	NP_001014589.1
2	rna-XM_008193309.2	170	155	79	458	680	658	NP_001096891.1
3	MSTRG.3723.4	0	0	0	720	517	511	NP_001096941.2
4	rna-XM_015979134.1	0	0	0	284	166	163	NP_001138180.1
5	rna-XM_015984327.1	0	0	0	95	92	277	NP_001163110.1
6	rna-XM_015984329.1	0	0	0	157	383	386	NP_001163110.1
7	rna-XM_015984330.1	0	0	0	184	238	398	NP_001163110.1
8	rna-XM_015984337.1	0	0	0	21	40	88	NP_001163110.1
9	rna-XM_015984332.1	0	0	0	145	165	375	NP_001163110.1
10	rna-XM_015984325.1	0	0	0	156	202	363	NP_001163110.1
11	rna-XM_015984333.1	0	0	0	183	185	372	NP_001163110.1
12	rna-XM_015984328.1	0	0	0	174	226	350	NP_001163110.1
13	rna-XM_015984798.1	14	17	6	48	102	73	NP_001163308.1
14	rna-XM_015982158.1	2	4	0	1500	424	1870	NP_001188635.1
15	rna-XM_015980425.1	0	0	0	41	110	498	NP_001188747.1
16	rna-XM_008202431.2	2780	2711	2396	3844	3177	4655	NP_001189037.1
17	MSTRG.6412.28	53	36	240	2933	1251	1475	NP_001245422.1
18	MSTRG.6412.22	939	746	557	6899	4020	3604	NP_001245422.1
19	rna-XM_008202703.2	437	407	272	902	1058	1033	NP_001245553.1
20	rna-XM_015985185.1	226	252	214	463	362	649	NP_001246155.1
21	MSTRG.2317.1	3	1	0	270	179	150	NP_001246377.1
22	rna-XM_015982118.1	0	0	0	7	137	32	NP_001246509.1
23	rna-XM_015977718.1	0	0	3	46	52	132	NP_001246539.1
24	MSTRG.3499.2	0	0	0	2347	3672	2835	NP_001246669.1
25	MSTRG.9139.2	0	0	0	383	24	499	NP_001246722.1
26	MSTRG.1955.1	46	269	63	1143	1216	1672	NP_001246787.1
27	rna-XM_008200355.2	0	0	0	156	165	1472	NP_001247202.1
28	rna-XM_008201549.2	892	774	884	1669	1406	2274	NP_001247335.1
29	MSTRG.2594.1	0	0	1	2069	1185	2292	NP_001259604.1
30	rna-XM_015979080.1	0	0	0	251	210	22	NP_001259788.1
31	MSTRG.4493.10	279	423	425	1221	1270	1173	NP_001260020.1
32	rna-XM_015979082.1	4	5	2	31	91	347	NP_001260068.1
33	MSTRG.3783.5	267	248	157	819	694	826	NP_001260243.1
34	MSTRG.9707.1	574	813	468	2081	1263	1893	NP_001261028.1
35	rna-XM_015978446.1	1020	920	754	1982	1727	3371	NP_001261081.1
36	MSTRG.3837.1	0	0	0	4865	4460	5462	NP_001261284.1
37	MSTRG.1478.9	0	0	0	194	175	207	NP_001262772.1
38	rna-XM_015980522.1	48	61	36	132	150	143	NP_001284821.1
39	MSTRG.3678.3	0	0	1	15	1181	2169	NP_001285180.1
40	rna-XM_008194983.2	704	394	682	11,275	11,691	14,844	NP_001287262.1

These count data indicate the number of sequence fragments that have been assigned to each gene. (n = 3, biological replication).

### Fine structure of the femur, trochanter, and coxa

**Figure 7:**
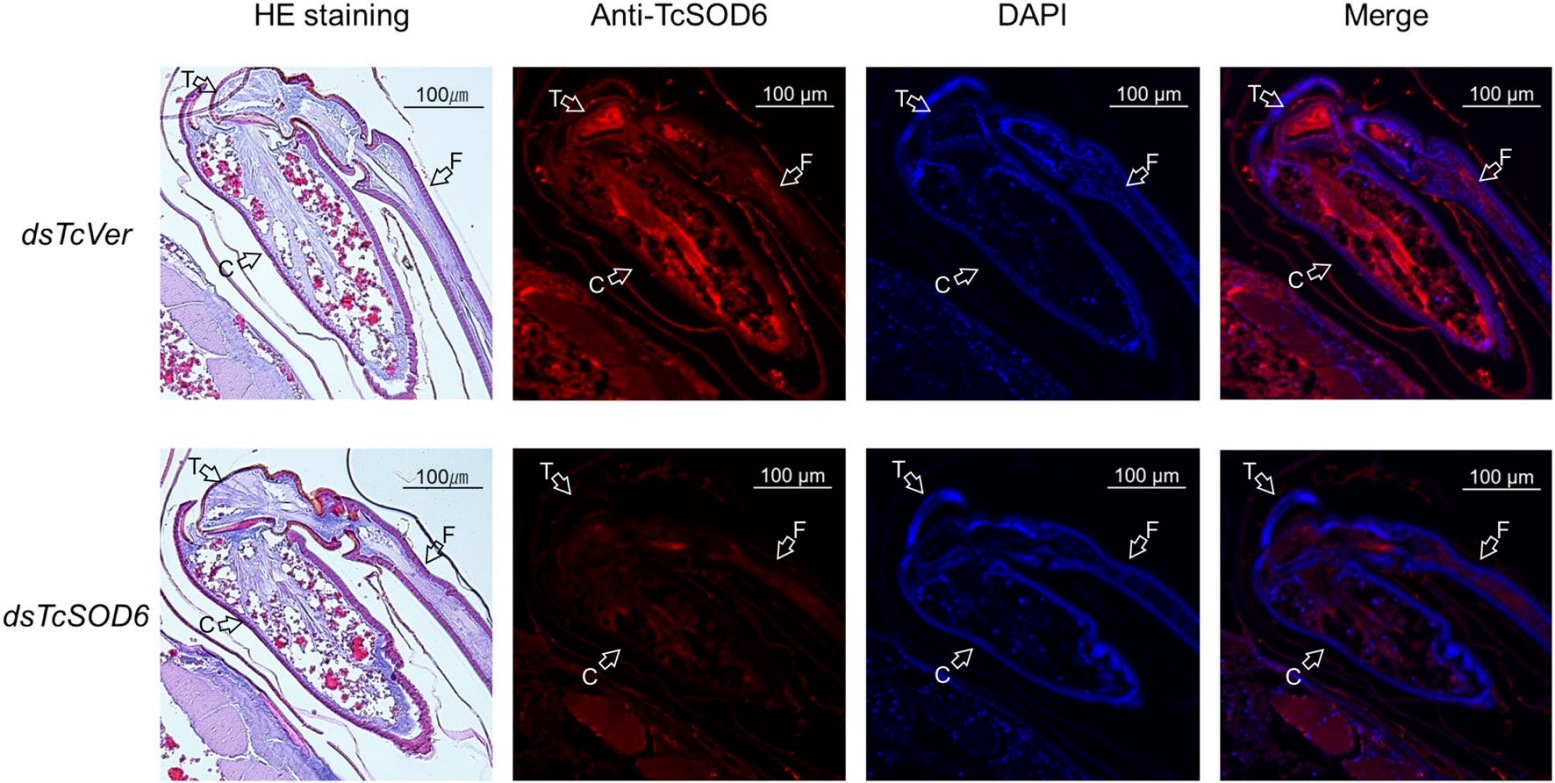
The fine structure of the femur, trochanter, and coxa in *TcVer-* and *TcSOD6*-knockdown hind legs. Representative polarized light microscopy and confocal laser scanning microscopy (× 100) images obtained from pupae at day 5 in the *TcVer-* and *TcSOD6-*knockdown groups. Upper panels indicate the *TcVer*-knockdown control group. Lower panels indicate the *TcSOD6-*knockdown group. TcSOD6 protein fluorescence is shown in red, and nuclear fluorescence is shown in blue. The structure of the hind legs was observed by visible light imaging of haematoxylin and eosin-stained sections. The open arrows indicate the different parts of the hind leg; C, coxa; T, trochanter; F, femur. Bar = 100 µm.

**Figure 8:**
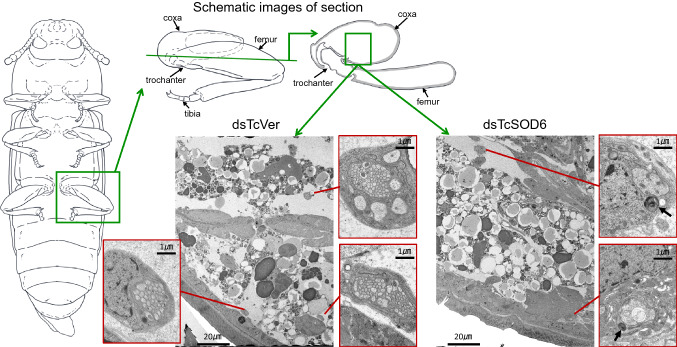
Ultrastructural images of coxa in *TcVer-* and *TcSOD6*-knockdown-hind legs. Representative transmission electron microscopy images obtained from day 6 pupae in the *TcVer*- and *TcSOD6*-knockdown groups. The positions of *T. castaneum* adult legs are shown in illustrations. The left panel shows a *TcVer*-knockdown pupa. Right panels show a *TcSOD6*-knockdown pupa. The inclusion bodies are indicated by a black arrow. The high magnification images are shown by the red boxes. Bar = 1 µm or 20 µm.

We examined the fine structure of the leg (femur, trochanter, and coxa) of *T. castaneum* by confocal laser scanning microscopy (Fig. 7). The TcSOD6 protein was mainly detected in the muscles, and the *TcSOD6*-knockdown group showed significantly reduced reactivity to an anti-TcSOD6 antibody relative to the *TcVer*-knockdown group in the femur, trochanter, and coxa (Fig. 7). Ultrastructural analysis by TEM revealed the inhibition of normal axon development in the nerve cells in the coxa of the *TcSOD6*-knockdown group. Agglomerates appearing to be protein aggregates (inclusion body) were observed (Fig. 8 [arrows] and Supplementary Figs. 5 and 6). Thus, these morphological changes suggested that knockdown of *TcSOD6* led to the inhibition of the development of properly movable legs during the pupal stage.

## Discussion

In this study, we investigated the function of *TcSOD6* using RNAi, transcriptome analysis, and histochemistry. Previous studies have shown that insect *SOD3* is more closely related to vertebrate *SOD1* than to vertebrate *SOD3*^5,17^. However, according to our new results, the cluster including *SOD6* was more closely related to vertebrate *SOD3* than to vertebrate *SOD1* or insect *SOD3*. Additionally, our phylogenetic analysis indicated that the *SOD6* cluster was limited to insect species. Phylogenetic analysis suggested that *SOD6* and vertebrate *SOD3* diverged from a part of *SOD5* lineage. We speculate that *SOD6* and vertebrate *SOD3* may have evolved from a common ancestral gene.

The TcSOD6 protein was found in the haemolymph, consistent with the presence of a predicted amino-terminal secretion signal peptide, suggesting that the TcSOD6 protein is secreted into the haemolymph. The highest *TcSOD6* mRNA expression level was measured on day 3 of the pupal stage, suggesting a potential function of *TcSOD6* in metamorphosis. When *TcSOD6* function during the pupal stage was investigated using RNAi, we found obvious phenotypic changes in the *TcSOD6*-knockdown adults. The *TcSOD6*-depleted beetles mainly displayed impaired movement of their middle and hind legs, which has not been previously reported in insects subjected to SOD gene knockdown. We initially speculated that the impaired leg movement resulted from tendinous dysfunction because the impairment of movement was restricted to the legs and the leg tendon is an endoskeleton structure providing attachment points for the muscles. However, there was no morphological change in the tendons in the *TcSOD6*-knockdown beetles. These findings indicate the occurrence of other mechanisms underlying the impaired leg movements. We additionally found that *TcSOD6*-knockdown insects exhibited a shorter lifespan and decreased amount of fat body relative to the controls. When the two knockdown groups were starved, the survival rate did not differ significantly between them, consistent with an inability to eat due to impaired leg mobility. Moreover, when we examined the movement of antennae and mouthparts in the *TcSOD6*-knockdown group, we did not find differences between the *TcSOD6*-knockdown and *TcVer*-knockdown control groups (Supplementary Videos 1–4).

We subsequently conducted a transcriptome analysis to identify possible differences between beetles with higher versus lower levels of *TcSOD6* mRNA during metamorphosis to investigate the potential molecular mechanisms underlying the impaired movement of the middle and hind legs. This analysis revealed that ten transcripts involved in neuronal development showed different levels after *TcSOD6*-knockdown. We further examined the cellular localization and function of these transcripts using the UniProt database (https://www.uniprot.org/). We focused on ankyrin 2, isoform N (UniProt: Q9NCP8), which was found to be upregulated upon *TcSOD6*-knockdown. The ankyrin 2 protein is distributed in the axons and dendrites of dopaminergic neurons^18^, where it is located in the plasma membrane, presynaptic membrane, and presynaptic active zone^18^. In addition, we found that several transcripts (sprint, isoform J; paralytic, isoform AN; sidestep, isoform D; and turtle, isoform I) were downregulated upon *TcSOD6*-knockdown (Tables 1 and 2). The sprint protein plays a role in axon extension and localizes to cortical cells and endocytic vesicles (UniProt: Q8MQW8)^19^, and the sidestep protein is involved in motoneuron axon guidance and localizes to cell membranes (UniProt: A0A0B4K6K0)^20–22^, whereas the turtle protein participates in axon guidance and localizes to cell membranes (UniProt: Q967D7)^23,24^. These findings suggest that the significantly downregulated transcripts may mediate axon extension and guidance functions affected by *TcSOD6*-knockdown. Based on the RNA-seq results following *TcSOD6*-knockdown, we further examined the fine structure of the middle legs, which revealed neuronal abnormality in the *TcSOD6*-knockdown group. Nerve cells were deformed, and normal axon development was inhibited in the *TcSOD6*-knockdown beetles. These observations were common to *TcSOD6*-knockdown beetles. Additionally, the observed fluorescence intensity indicated strong TcSOD6 protein expression in the adult muscles. These findings indicate that the downregulation of *TcSOD6* may affect some of the processes for larval neurons are remodelled to new adult functions in the legs during metamorphosis.

Holometabolous insects undergo a dramatic change in body structure in the pupal development, including neuronal remodelling. The neuronal remodelling process in the ventral ganglia of *Manduca sexta* involves two main processes: programmed cell death in larval-specific neurons and neuron remodelling for the maturation of imaginal neurons^7^. In larval motoneurons, programmed cell death is observed only in some larval cells, as most of these cells survive^7,25^. Larval motoneuron cells are remodelled in adults without the differentiation of new motoneuron cells during pupal development^25,26^. The motor neurons that undergo large-scale reorganization of their neuronal connections during metamorphosis are reused in adults^25^. At this metamorphosis stage, the degeneration of dendrites and the formation of new dendrites occur. The muscular system is also remodelled during metamorphosis, as larval muscles undergo programmed cell death^25,27^. All larval muscles degenerate, and adult muscles are formed via the proliferation and differentiation of primordia present in the larval body^27^. During this process, adult motoneurons are attached to newly developing adult muscles^25^. We speculate that a defect in *TcSOD6* expression might lead to abnormal motoneuron remodelling, resulting in the incorrect innervation of adult leg motoneurons.

As ROS production increases during the pupal stage in the holometabolous insect *B. mori*^9^, *T. castaneum* pupae might show a similar increase in ROS during metamorphosis. We speculated that *TcSOD6*-downregulation may more strongly increase the levels of ROS in the pupal stage than observed in *B. mori*. Consequently, the expression of neurodevelopment-related transcripts might be secondarily induced by ROS. These findings suggest that *TcSOD6* is required during metamorphosis for the development of properly movable legs. However, we cannot elucidate why abnormal movement was limited to only their legs in this study. Therefore, our results suggest that some systems cannot be explained only by the regulatory relationship between ROS and *TcSOD6*. In future studies, we will investigate the mechanism by which the TcSOD6 protein controls the neurodevelopment-related genes identified in this study.

In conclusion, we identified a novel function of insect SOD6 in controlling the development of properly movable legs during metamorphosis, which might be indirectly mediated by controlling ROS levels and neurodevelopment-related gene expression. These findings provide novel insights into the function of insect SOD6 during metamorphosis.

## Methods

### Insects

The *T. castaneum* GA-1 strain was used in all experiments. Insects were reared on whole wheat flour containing 5% brewer’s yeast^28^ and maintained at 27 °C under a 16-h light/8-h dark cycle.

### Obtaining TcSOD sequences and bioinformatic analysis

TcSOD sequences were obtained from BeetleBase (Supplementary Table 1) in our previous study^6^. A search for *SOD* orthologues in other insect species was conducted using BLAST methods. These SOD sequences were obtained from the NCBI (http://www.ncbi.nlm.nih.gov) or DDBJ (https://www.ddbj.nig.ac.jp/index-e.html) database. A global homology search was conducted using Genetyx ver. 11 (Genetyx Co. Ltd., Tokyo, Japan). A protein motif analysis was performed by using SMART (http://smart.embl-heidelberg.de/). The alignment analysis was performed using Genetyx ver. 11 (Genetyx Co. Ltd., Tokyo, Japan) with CLUSTAL 2.1 Multiple Sequence Alignments^29^. Phylogenetic trees were generated using GENETYX-Tree 2.2.0 (Genetyx Co. Ltd., Tokyo, Japan).

### Purification of total RNA and cDNA synthesis from whole-body samples

Total RNA from each developmental stage was isolated and purified from the whole bodies of *T. castaneum*. Thirty eggs were used as one embryonic stage sample, and individuals were used as the samples from other stages. Three replications of total RNA from each developmental stage were stored at − 80 °C until use. Whole bodies were homogenized with TRIzol™ Reagent (Life Technologies, Carlsbad, CA, USA) and processed for RNA purification in accordance with the manufacturer’s instructions. Total RNA (1 μg) was treated with deoxyribonuclease I, amplification grade (Life Technologies). Thereafter, 500 ng of DNase-treated total RNA was employed as a template for cDNA synthesis using the PrimeScript™ 1st strand cDNA Synthesis Kit (Takara Bio, Inc., Kusatsu, Shiga, Japan). cDNA cloning was performed using specific primers (Supplementary Table 5).

Quantitative real-time PCR (RT–qPCR) was performed in a 20 μL reaction volume containing 0.125 μL of cDNA template and specific primers (Supplementary Table 6) with KAPA SYBR® FAST qPCR Kit Master Mix (2×) ABI Prism™ (Sigma–Aldrich Corporation, St. Louis, MO, USA), in accordance with the manufacturer’s instructions, on a StepOnePlus™ Real-Time PCR System (Applied Biosystems, Carlsbad, CA, USA). Relative gene expression levels were calculated using the 2^−∆∆Ct^ method, with the *T. castaneum* ribosomal protein S6 gene (*RpS6*, gene identification [ID] number 288869507) as an endogenous reference for the standardization of RNA expression levels. All data were calibrated against universal reference data. Relative expression levels against a reference sample are represented as relative quantification (RQ) values. All samples were assayed with three biological replications. Statistical significance was determined by a two-tailed Student’s t-test using Excel (Microsoft, Redmond, WA, USA). The *P* values of < 0.05 were significant.

### Immunoblotting

The custom-made rabbit polyclonal antiserum against TcSOD6 was raised against the synthetic peptide LNVDPASSPRTYH (corresponding to amino acid positions 726–738 in TcSOD6; Merck, Sigma–Aldrich Co. Ltd., Darmstadt, Germany).

Haemolymph was collected from *T. castaneum* pupae (n = 10; 6 days after dsRNA injection) according to the methods described by Tabunoki et al.^30^ Pupal whole bodies (n = 5; 6 days after dsRNA injection) were homogenized with an EzRIPA lysis buffer kit (ATTO Co. Ltd, Tokyo, Japan) and processed for protein extraction according to the manufacturer’s instructions. The protein concentration was determined by using a BCA protein assay kit (Thermo Scientific Co., Ltd., Rock-ford, IL). Recombinant TcSOD6 (rTcSOD6) protein was prepared using the ProCubeTMTb custom recombinant protein synthesis and purifying service (Sysmex Co. Ltd., Hyogo, Kobe, Japan). The amino acid sequence of the *TcSOD6* ORF without the predicted secreted signal sequence was ligated (19E-1122L,127.1 KDa) into the baculoviral transfer pHS13a vector, and the *TcSOD6* nucleotide sequence was verified by sequencing using a 3500 Genetic Analyzer (Applied Biosystems Co. ltd.). The TcSOD6-pHS13a vector was used for homologous recombination with the BmNPV genome. Recombinant BmNPV-TcSOD6 was used to infect *B. mori* pupae, and the infected pupae were homogenized with phosphate-buffered saline (PBS), pH 7.4, with cOmplete™ Protease Inhibitor Cocktail Tablets EDTA-free (Roche Diagnostics K.K. Grenzach-Wyhlen, Germany) and 1% (w/v) Triton X-100 (Sigma-Ardlich). The homogenate was centrifuged at 100,000×*g* for 60 min at 4 °C, and the supernatant was collected. The supernatant was filtered by Minisart (Sartrius co. Ltd., Tokyo, Japan). The filtrated supernatant, including the rTcSOD6 protein, was purified by open column chromatography using a DDDDK-tagged protein purification gel (MBL Co. Ltd., Tokyo, Japan). The rTcSOD6 protein was eluted with PBS, pH 7.4, containing 0.1% (w/v) Triton X 100 and 0.1 mg/mL DYKDDDDK peptide.

Protein samples (12.5 µg/lane), the rTcSOD6 protein (0.55 µg/lane), and the Mark12™ Unstained Standard (Thermo Scientific Co., Ltd., Rockford, IL) were separated by SDS–PAGE and transferred to a polyvinylidene difluoride membrane. Nonspecific binding was blocked with EzBlock Chemi (ATTO Co. Ltd, Tokyo, Japan) with 3% BSA for 1 h at 37 °C, and the membrane was then incubated with the rabbit anti-TcSOD6 serum (1:1000; custom made by Merck, Sigma–Aldrich Co. Ltd.) overnight at 4 °C. After washing, the membrane was incubated with a goat anti-rabbit IgG (H + L) antibody conjugated to horseradish peroxidase (HRP) conjugate at 1:500,000 (SA00001-2; Proteintech Inc, Rosemont, IL, USA) for 1 h at room temperature. Membranes were developed using a chemiluminescent substrate (Clarity Western ECL Substrate; Bio-Rad Laboratories, Inc., Hercules, CA, USA).

### Synthesis and injection of dsRNA

The E-RNAi web service^31^ (http://www.dkfz.de/signalling/e-rnai3/) was used to evaluate the possible off-target effects of dsRNA. *TcSOD6* dsRNA was synthesized from 369 bp within the target site and was amplified by PCR using the *T. castaneum* pupal cDNA library. The primers used for PCR amplification are listed in Supplementary Table 7. The resulting fragment was ligated into a vector using a TOPO® TA Cloning® Kit for Subcloning (450641, Thermo Fisher Scientific, Waltham, MA, USA), cloned with Competent Quick DH5α cells (Toyobo Co., Ltd., Tokyo, Japan), and sequenced. *TcVer* (GenBank AY052390) was used as a negative control. *TcVer* dsRNA was synthesized according to the methods described by Arakane et al.^32^ dsRNA for each target was synthesized with the T7 RiboMAX™ Express RNAi System (Promega Corporation, Madison, WI, USA) in accordance with the manufacturer’s protocols. *Tribolium castaneum* pharate pupae (1 day before the pupal molt) or 0-day adults were injected with dsRNA (600 ng/200 nL/body) using a microinjection system (Narishige Co. Ltd. Tokyo, Japan) under a stereomicroscope. Total RNA was isolated from 4-day-old pupae (5 days after dsRNA injection) to analyse the transcript levels. RT–qPCR was performed with the specific primers listed in Supplementary Table 6 to assess the knockdown efficiency of the target genes. In addition, the phenotype of each insect group was investigated. Adult survival was calculated as the number of live insects over a period of 50 days starting from adult day 0. Adult leg movements were recorded by digital light microscopy (KEYENCE, Osaka, Japan). *Tribolium castaneum* adults injected with dsRNA at the pharate pupal stage were examined at adult eclosion (8 days after injection), and adults injected with dsRNA immediately after adult eclosion were examined as 5-day-old adults.

### Histochemistry

The middle and hind legs of 1-day-old adults (8 days after the injection of *TcSOD6* or *TcVer* dsRNA) were dissected and fixed with 4% paraformaldehyde (until the legs sank to the bottom of the tube). To remove tissues, the samples were treated overnight with 10 M NaOH at 95 °C and then washed three times with PBS. Each sample was stained with fluorescein-conjugated chitin-binding domain (FITC-CBD) probes (dilution, 1:3000 in PBS; New England Biolabs, Ipswich, MA, USA) and incubated at room temperature for 3 h. After washing off the excess probes, the tissues were observed under a Leica M165 FC stereomicroscope equipped with appropriate filters^33^.

Five-day-old pupae (6 days after the injection of *TcSOD6* or *TcVer* dsRNA) were dissected and fixed with 4% paraformaldehyde and embedded in paraffin. After deparaffinization with xylene, the sections were blocked with 0.5% casein/Tris-saline (150 mM NaCl/10 mM Tris–HCl, pH 7.6) at room temperature for 1 h and then incubated with the primary antibody (Custom rabbit anti-TcSOD6 serum (Sigma–Aldrich Co. Ltd. St. Louis, MO, USA) diluted 1:100 with 0.5% casein/Tris-saline) at 4 °C overnight. After washing with Tris-saline, these sections were incubated with a mixture of rhodamine-labelled anti-rabbit immunoglobulin (Merck Millipore, × 100 dilution) and FITC-labelled anti-mouse immunoglobulin (Merck Millipore, × 100 dilution) at 37 °C for 1 h. Counterstaining was performed with DAPI (Merck Millipore, × 1000 dilution), and images were then acquired with an LSM710 confocal laser scanning microscope (Carl Zeiss Co. Ltd., Munich, Germany).

Additionally, deparaffinized sections were stained with haematoxylin–eosin (HE), and images were acquired with a BX-51P polarized light microscope (Olympus Corporation, Tokyo, Japan).

### Transmission electron microscopy analysis

One-day-old adults (8 days after injection dsRNA for *TcSOD6* or *TcVer*) were fixed with 2% paraformaldehyde (PFA) and 2% glutaraldehyde (GA) in 0.1 M cacodylate buffer, pH 7.4, at 4 °C overnight. The fixed samples were washed 3 times with 0.1 M cacodylate buffer for 30 min each and were then postfixed with 2% osmium tetroxide (OsCh) in 0.1 M cacodylate buffer at 4 °C for 3 h, afater which the samples were dehydrated in graded ethanol solutions (50%, 70%, 90%, anhydrous). Specifically, the samples were incubated in 50% and 70% ethanolfor 30 min each at 4 °C, 90% ethanol for 30 min at room temperature, and 3 changes of anhydrous ethanol for 30 min each at room temperature. After these dehydration steps, the samples were continuously dehydrated in anhydrous ethanol at room temperature overnight.

Then, the samples were infiltrated with propylene oxide (PO) 2 times for 30 min each and placed into a 70:30 mixture of PO and resin (Quetol-812; Nisshin EM Co., Tokyo, Japan) for 1 h. Then, the cap of the tube was left open, and PO was volatilized overnight.

Embedding and polymerization were conducted as follows: the samples were transferred to fresh 100% resin and polymerized at 60 °C for 48 h. Subsequently, we obtained ultrathin sections as follows: 80 nm ultrathin sections of the polymerized resins were cut with a diamond knife using an ultramicrotome (Ultracut UCT; Leica, Vienna, Austria), and the sections were mounted on copper grids. They were then stained with 2% uranyl acetate at room temperature for 15 min and washed with distilled water, followed by secondary staining with a lead staining solution (Sigma–Aldrich Co., Tokyo, Japan) at room temperature for 3 min. The grids were observed under a transmission electron microscope (JEM-1400Plus; JEOL Ltd., Tokyo, Japan) at an acceleration voltage of 100 kV. Digital images (3296 × 2472 pixels) were obtained with a CCD camera (EM-14830RUBY2; JEOL Ltd., Tokyo, Japan).

### RNA-seq analysis

Three total RNA samples were used for the RNA-seq analysis of *TcVer-* or *TcSOD6*-knockdown pupae 5 days after dsRNA injection and 4 days after pupal eclosion. RNA quality was assessed using TapeStation 2200 software (Agilent Technologies, Inc., Santa Clara, CA, USA). Additionally, cDNA libraries for paired-end sequencing were constructed with 100 ng of total RNA from the ds*TcVer* and ds*TcSOD6* groups (n = 3 each) and a NovaSeq® 6000 SP Reagent Kit (Illumina, Inc., San Diego, CA, USA) accoridng to the manufacturer’s instructions. The libraries were sequenced (101 bp, paired-end) on the Illumina NovaSeq6000 platform, and FASTQ files were assessed with the Trim Galore! (v0.4.5) trimming tool (https://www.bioinformatics.babraham.ac.uk/projects/trim_galore/). The *T. castaneum* genome (GCF_000002335.3) sequence was retrieved from the NCBI Genome database (https://www.ncbi.nlm.nih.gov/assembly/GCF_000002335.3). The obtained FASTQ sequence files were aligned to the genomic reference sequence using the HISAT2 v2.1.0 alignment program for mapping RNA-seq reads with the default parameters^34^. Next, the obtained SAM files were converted to BAM files with SAMtools v1.8^35^. Transcript abundance was estimated using the StringTie v1.3.4 assembler, and the count data were extracted with the Subread v1.6.0 read aligner^36,37^. All statistical analyses were performed using R software version 3.4.3 (https://www.r-project.org). The TCC and DEseq2 packages were used to normalize the data and to compare the *TcVer*- and *TcSOD6*-knockdown groups^38^. An MA plot was generated using TIBCO Spotfire Desktop v7.6.0 with the “Better World” program licence (TIBCO Software, Inc., Palo Alto, CA, USA; http://spotfire.tibco.com/better-world-donation-program/).

### Gene enrichment and molecular interaction analyses

Gene enrichment analysis was performed using the Metascape gene annotation and analysis resource^39^ (http://metascape.org/). A gene list for Metascape analysis was generated from the TCC output. The gene ID numbers were converted from the *T. castaneum* RNA-Seq data to *D. melanogaster* NCBI ID numbers for the construction of an assignment table. Then, the list of genes obtained from the RNA-Seq data was input into the IntAct Molecular Interaction Database^40^ to identify significant molecular interactions.

## Supplementary Information

Supplementary Video 1.

Supplementary Video 2.

Supplementary Video 3.

Supplementary Video 4.

Supplementary Information.

## Data Availability

The RNA sequencing datasets generated and/or analysed during the current study are available in the Sequence Read Archive, DNA Data Bank of Japan repository, under the following accession IDs: *TcVer*-knockdown groups (SRA accession numbers: DRR232570, DRR232571, and DRR232572) and *TcSOD6*-knockdown groups (DRR232567, DRR232568, and DRR232569). The nucleotide sequence reported in this paper has been submitted to the GenBank/ DNA Data Bank of Japan repository, SAKURA data bank under accession number LC430326.

## References

[1] Cadenas E, Ahmad S (1995). Mechanisms of oxygen activation and reactive oxygen species detoxification. Oxidative Stress and Antioxidant Defenses in Biology.

[2] Korsloot A, van Gestel CAM, van Straalen NM (2004). Environmental Stress and Cellular Response in Arthropods.

[3] Ahmad S, Ahmad S (1995). Antioxidant mechanisms of enzymes and proteins. Oxidative Stress and Antioxidant Defenses in Biology.

[4] Miller AF (2012). Superoxide dismutases: Ancient enzymes and new insights. FEBS Lett..

[5] Landis GN, Tower J (2005). Superoxide dismutase evolution and life span regulation. Mech. Ageing Dev..

[6] Kobayashi Y (2019). Comparative analysis of seven types of superoxide dismutases for their ability to respond to oxidative stress in *Bombyx mori*. Sci. Rep..

[7] Gilbert L, Tata J, Atkinson B (1996). Metamorphosis: Postembryonic Reprogramming of Gene Expression in Amphibian and Insect Cells.

[8] Nojima Y (2015). Superoxide dismutases, SOD1 and SOD2, play a distinct role in the fat body during pupation in silkworm *Bombyx mori*. PLoS ONE.

[9] Nojima Y (2019). Superoxide dismutase down-regulation and the oxidative stress is required to initiate pupation in *Bombyx mori*. Sci. Rep..

[10] Herndon N (2020). Enhanced genome assembly and a new official gene set for *Tribolium castaneum*. BMC Genom..

[11] Dönitz J, Gerischer L, Hahnke S, Pfeiffer S, Bucher G (2018). Expanded and updated data and a query pipeline for iBeetle-Base. Nucleic Acids Res..

[12] Tomoyasu Y, Denell RE (2004). Larval RNAi in Tribolium (Coleoptera) for analyzing adult development. Dev. Genes Evol..

[13] Bucher G, Scholtenn J, Klingler M (2002). Parental RNAi in Tribolium (Coleoptera). Curr. Biol..

[14] Ding F, Dokholyan NV (2008). Dynamical roles of metal ions and the disulfide bond in Cu, Zn superoxide dismutase folding and aggregation. Proc. Natl. Acad. Sci. U.S.A..

[15] Perry JJP, Shin DS, Getzoff ED, Tainer JA (2010). The structural biochemistry of the superoxide dismutases. Biochim. Biophys. Acta.

[16] Shin DS (2009). Superoxide dismutase from the eukaryotic thermophile *Alvinella pompejana*: Structures, stability, mechanism, and insights into amyotrophic lateral sclerosis. J. Mol. Biol..

[17] Colinet D, Cazes D, Belghazi M, Gatti JL, Poirié M (2011). Extracellular superoxide dismutase in insects: Characterization, function, and interspecific variation in parasitoid wasp venom. J. Biol. Chem..

[18] Yamamoto M, Ueda R, Takahashi K, Saigo K, Uemura T (2006). Control of axonal sprouting and dendrite branching by the Nrg–Ank complex at the neuron-glia interface. Curr. Biol..

[19] Sakuma C (2014). *Drosophila* Strip serves as a platform for early endosome organization during axon elongation. Nat. Commun..

[20] Sink H, Rehm EJ, Richstone L, Bulls YM, Goodman CS (2001). Sidestep encodes a target-derived attractant essential for motor axon guidance in *Drosophila*. Cell.

[21] Meyer F, Aberle H (2006). At the next stop sign turn right: the metalloprotease Tolloid-related 1 controls defasciculation of motor axons in *Drosophila*. Development.

[22] Siebert M, Banovic D, Goellner B, Aberle H (2009). *Drosophila* motor axons recognize and follow a sidestep-labeled substrate pathway to reach their target fields. Genes Dev..

[23] Al-Anzi B, Wyman RJ (2009). The *Drosophila* immunoglobulin gene turtle encodes guidance molecules involved in axon pathfinding. Neural Dev..

[24] Bodily KD, Morrison CM, Renden RB, Broadie K (2001). A novel member of the Ig superfamily, turtle, is a CNS-specific protein required for coordinated motor control. J. Neurosci..

[25] Tissot M, Stocker RF (2000). Metamorphosis in *Drosophila* and other insects: The fate of neurons throughout the stages. Prog. Neurobiol..

[26] Tsujimura H (1989). Metamorphosis of wing motor system in the silk moth, *Bombyx mori*: Origin of wing motor neurons. (flight motor neuron/insect/neural development). Dev. Growth Differ..

[27] Tshujimura H (1983). Development of neural innervation of the indirect flight muscles during metamorphosis in *Bombyx mori*. Zool. Mag..

[28] Lasley EL (1960). Tribolium Information Bulletin No. 3.3-13.

[29] Larkin MA (2007). ClustalW and Clustal X version 2.0. Bioinformatics.

[30] Tabunoki H, Dittmer NT, Gorman MJ, Kanost MR (2019). Development of a new method for collecting hemolymph and measuring phenoloxidase activity in *Tribolium castaneum*. BMC Res. Notes.

[31] Horn T, Boutros M (2010). E-RNAi: A web application for the multi-species design of RNAi reagents–2010 update. Nucleic Acids Res..

[32] Arakane Y (2009). Molecular and functional analyses of amino acid decarboxylases involved in cuticle tanning in *Tribolium castaneum*. J. Biol. Chem..

[33] Arakane Y (2011). Both UDP N-acetylglucosamine pyrophosphorylases of *Tribolium castaneum* are critical for molting, survival and fecundity. Insect Biochem. Mol. Biol..

[34] Kim D, Paggi JM, Park C, Bennett C, Salzberg SL (2019). Graph-based genome alignment and genotyping with HISAT2 and HISAT-genotype. Nat. Biotechnol..

[35] Li H (2009). The sequence alignment/map format and SAMtools. Bioinformatics.

[36] Liao Y, Smyth GK, Shi W (2013). The Subread aligner: Fast, accurate and scalable read mapping by seed-and-vote. Nucleic Acids Res..

[37] Liao Y, Smyth GK, Shi W (2013). FeatureCounts: An efficient general purpose program for assigning sequence reads to genomic features. Bioinformatics.

[38] Sun J, Nishiyama T, Shimizu K, Kadota K (2013). TCC: An R package for comparing tag count data with robust normalization strategies. BMC Bioinform..

[39] Zhou Y (2019). Metascape provides a biologist-oriented resource for the analysis of systems-level datasets. Nat. Commun..

[40] Orchard S (2014). The MIntAct project–IntAct as a common curation platform for 11 molecular interaction databases. Nucleic Acids Res..

