# Supplementary material for: Superoxide dismutase 6 is required during metamorphosis for the development of properly movable legs in Tribolium castaneum
Source: Sci Rep. 2022 Apr 27;12:6900. doi: 10.1038/s41598-022-10166-3 (PMC9046187; doi:10.1038/s41598-022-10166-3)
Supplement: Supplementary file 5 — Supplementary Information. [file 41598_2022_10166_MOESM5_ESM.pdf]

Supplementary Materials

Superoxide dismutase 6 is required during metamorphosis for the development of properly movable legs in *Tribolium castaneum*

Maaya Nishiko, Takuma Sakamoto, Seulgi Mun, Mi Young Noh, Yasuyuki Arakane, Michael R. Kanost, Katsuhiko Arai, and Hiroko Tabunoki*

*Corresponding author. Email: h_tabuno@cc.tuat.ac.jp

This PDF file includes:

Supplementary Figure 1. Comparison of *T. castaneum* SOD6 amino acid sequences obtained from BeetleBase and translated via cDNA cloning.
Supplementary Figure 2. Alignment of *T. castaneum* SOD6 of three SOD_Cu/Zn domains with other SOD6 proteins.
Supplementary Figure 3. Verification of decreased *TcSOD6* mRNA expression in *TcSOD6* knockdown pupae.
Supplementary Figure 4. Full image for figure.4 a: SDS-PAGE, b: Immunoblot
Supplementary Figure 5. Ultrastructural images of coxa in *TcVer*-and *TcSOD6*-knockdown hind legs in biological replicant 2.
Supplementary Figure 6. Ultrastructural images of coxa in *TcVer*-and *TcSOD6*-knockdown hind legs in biological replicant 3.
Supplementary Video 1. *TcSOD6*-knockdown insect movement after dsRNA injection in the prepupa.
Supplementary Video 2. *TcVer*-knockdown insect movement after dsRNA injection in the prepupa.
Supplementary Video 3. *TcSOD6*-knockdown insect movement following dsRNA injection immediately after adult eclosion.
Supplementary Video 4 Video. *TcVer*-knockdown insect movement following dsRNA injection immediately after adult eclosion.
Supplementary Table 1. SOD sequence IDs obtained from BeetleBase.
Supplementary Table 2. Gene IDs included the phylogenic tree in this study.
Supplementary Table 3. *TcSOD6* primers used for cDNA cloning.
Supplementary Table 4. *TcSODs* primers used for quantitative reverse transcription PCR.
Supplementary Table 5. Primers used for dsRNA synthesis.

```
TcSOD6_cloning    1 MGQIIFVWFILLKLATSLELKSYVSDHGLHGTITFRKIGPEIKIITDLNATLEYPNQVWSWAVVEFPVDYTELENRCWESKLGRELVNLDTIYGPLVLPD 100
TC011770-RA       1 ......................................................................S............................. 100

TcSOD6_cloning  101 NGTSEFSTQELQINGDVGIYGKSLLFKDPETNTRICATITMVDKAQEKIAVARFNSPIAGSVHFRWFSTKDNHNDMVVTTDLYHVSNKENHSQSLDFTEH 200
TC011770-RA     101 .............................................................I...................................... 200

TcSOD6_cloning  201 LWKIYATDILDSDKERPDCNFLQLVFDPDNTGDQALGDLDSRLGKIKISTDYKRKKFKTLFTDQRLILLPSDLQGPQRSLYLVIFERKHPDSFLACAKIR 300
TC011770-RA     201 .................................................................................................... 300

TcSOD6_cloning  301 YERPINAKVIIQSGGIKGEVRMTQFSKFEPTFVNFDLTTARGDLETRLVYSSSVAGYKIHELPVKPSKTLGQNENSCLTTKFVYNPVKIDPNVAPPSGLG 400
TC011770-RA     301 ...................................................................I................................ 400

TcSOD6_cloning  401 TQNQYLVGDLSGKLQGRNNQTVLVPTGHELSGLYWDTLLPLQGKFSIIHRTLVIYKNTQYPKGEILSEPWICGGIALYEPNFRYQKPIFTAEVIFRYPTV 500
TC011770-RA     401 .....................A.............................................................................. 500

TcSOD6_cloning  501 GRVLMRQVRDEPWSDTSILVEYLVHADGAALNNSKDHRWAIHEEPPGKDFYSWKERCLSSGQIYNPYKIDALDACTGGLCRVGDLTARLGTIEISGKIAD 600
TC011770-RA     501 ...........................................................................................V........ 600

TcSOD6_cloning  601 SDRVSRTMWTDPFVPLTGQFSVLGKSLVVYDDFGPKARGERLACSIIGGVYRRKAVVKDWFPNGVPISVKGKIEFYQQTEYDITNVEVNLQGLSDNSGYH 700
TC011770-RA     601 ....................................................C............................................... 700

TcSOD6_cloning  701 IHITPVEENLEFPCEASSLYDHWNPLNVDPASSPRTYHGTPDQYEMGDLSGKFGTLDNQTYYKTDYNDTMLPLFGPKSIVGRSIIIHKK-DTSRWACATI 799
TC011770-RA     701 .........................................................................................V.......... 800

TcSOD6_cloning  800 ERGYSPSEARELRAIASFHHPDGYAYGYLKMKQLIYNDGSKSDTTIEVKLRHPGKNDRNVTRDHDWAIYVNPVGVDAAVKTQNTRCVAGGYIWNPYFTQL 899
TC011770-RA     801 .................................................................................................... 900

TcSOD6_cloning  900 ADPLNGDLYRQECGPENPLRCHVGDLSARLGTIDIGLQRKVFTDANFPLEGDVTAIGRSIVIMSPNRRPERYACANIEPDYDIIKYANIEKPPRFVLSQF 999
TC011770-RA     901 .................................................................................................... 1000

TcSOD6_cloning 1000 IEEVREILGIPDWFLTVDSRKTKSLHSDACVQLLLHFKGPLANRLEQDFSRLLASGRLDTPTLYIPGFINTKRKVKIAYKQCGVQDPNEKSKKRTGFFFA 1099
TC011770-RA    1001 ..........................................................................................---------- 1090

TcSOD6_cloning 1100 RSGSNPAKFAHSLIVVIVLSYLL                                                                              1122
TC011770-RA    1091 .--....................                                                                              1111
```

Supplementary Figure 1. Comparison of *T. castaneum* SOD6 amino acid sequences obtained from BeetleBase and translated via cDNA cloning. *TcSOD6* amino acid sequences obtained from the BeetleBase and cDNA cloning were aligned and compared. Different amino acid sequences are shown the black-colored.

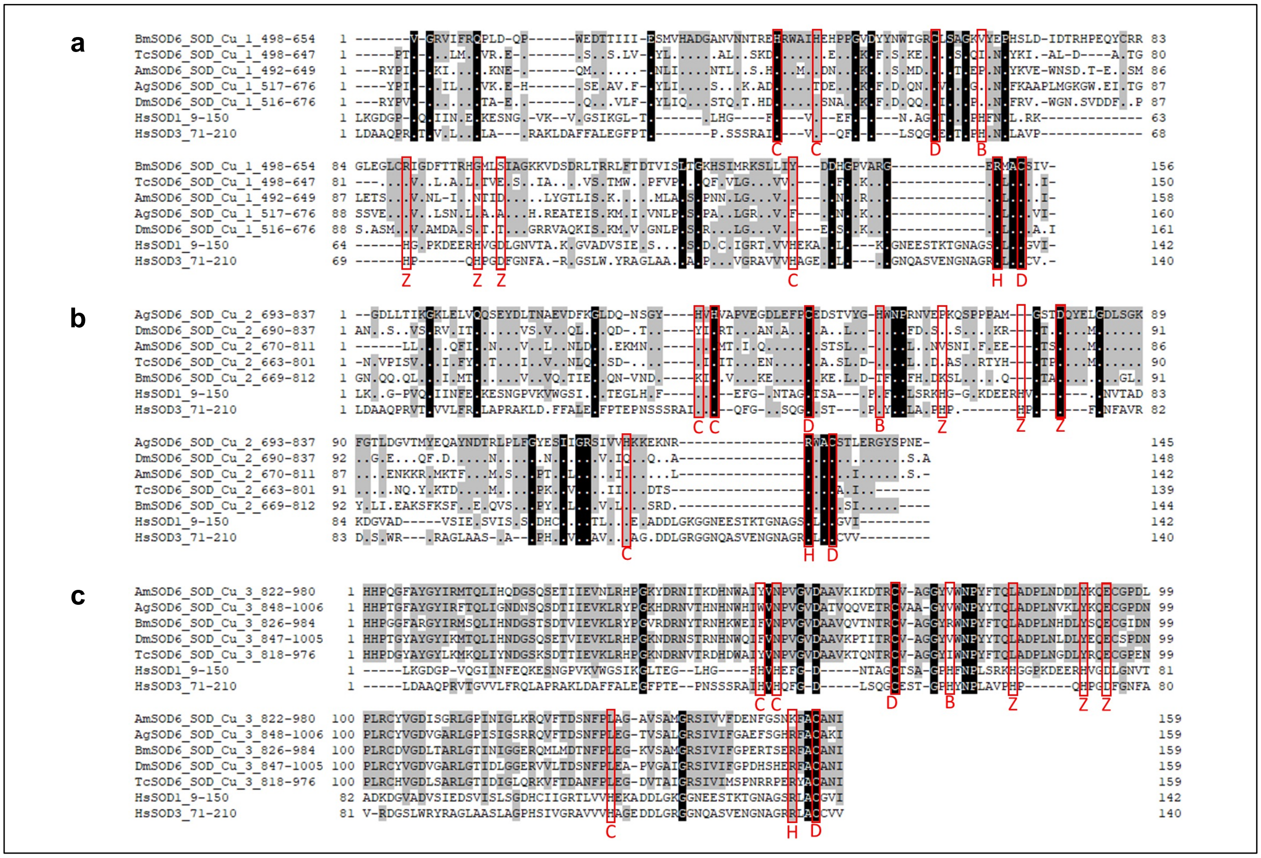

Supplementary Figure 2. Alignment of *T. castaneum* SOD6 of three SOD_Cu/Zn domains with other SOD6 proteins. The alignment of amino acid sequences for three SOD_Cu/Zn domains in insect SOD6. (A) The first SOD_Cu/Zn domain corresponds to the N-terminal side of the SOD_Cu/Zn domain in *T. castaneum*. (B) The second SOD_Cu/Zn domain corresponds to the middle side of the SOD_Cu/Zn domain in *T. castaneum*. (C) The third SOD_Cu/Zn domain corresponds to the C-terminal side of the SOD_Cu/Zn domain in *T. castaneum*. The conserved C, copper-binding amino acids; D, disulfide cysteine; B, bridging histidine; Z, zinc-binding ligand; H, H_2O_2 ligands residue are marked by red characters, while an open square indicates these conserved amino acid residues among species.

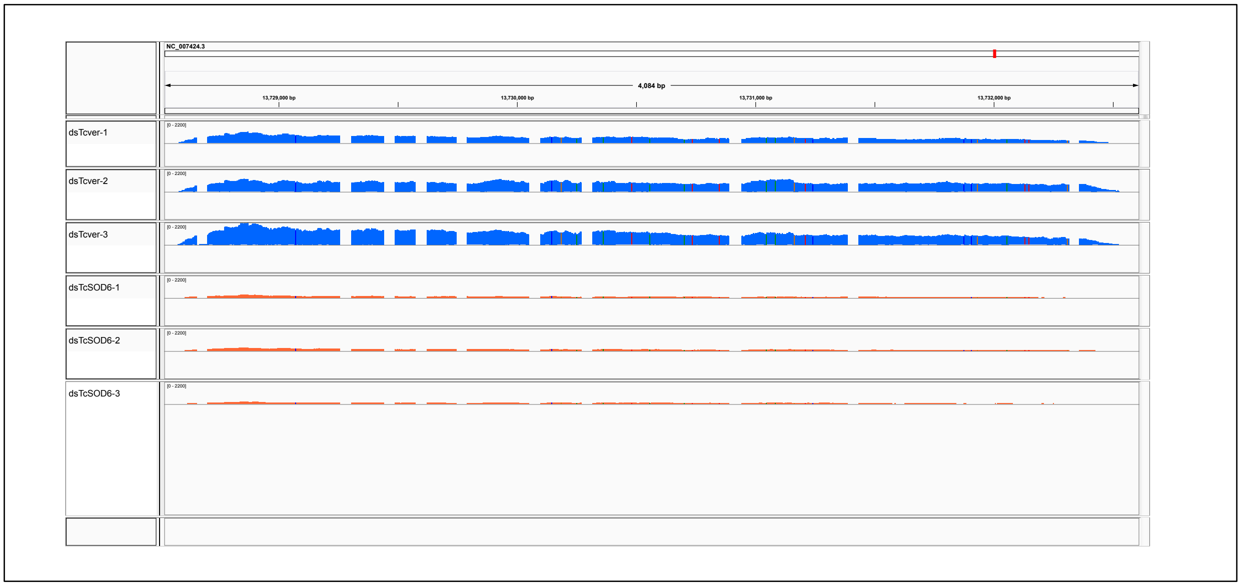

Supplementary Figure 3. Verification of decreased *TcSOD6* mRNA expression in *TcSOD6*-knockdown pupae. Verification of *TcSOD6* mRNA expression by RNA-seq analysis. Histograms show the frequency of *TcSOD6* between *TcVer*- and *TcSOD6*-knockdown groups (n=3, respectively).

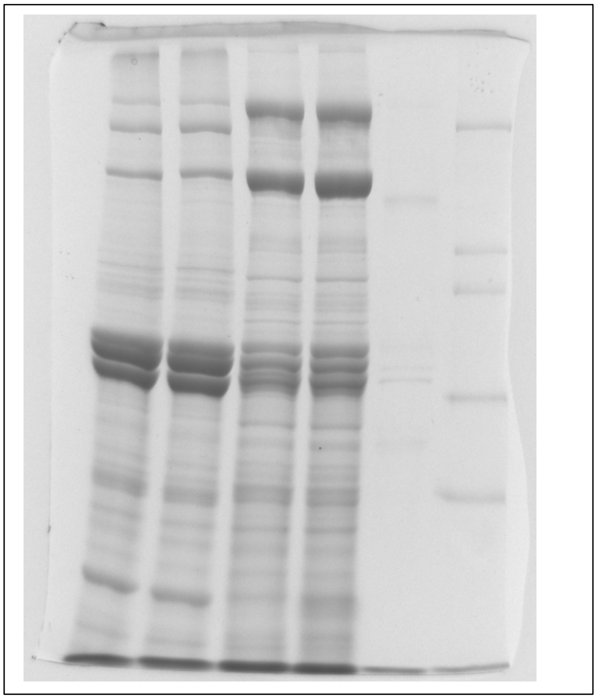

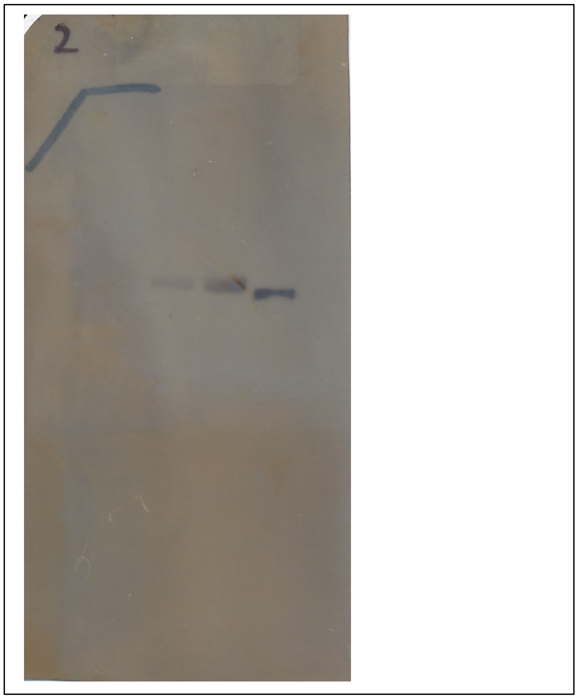

Supplementary Figure 4. Full image for Figure 4. a, SDS-PAGE, b, Immunoblot

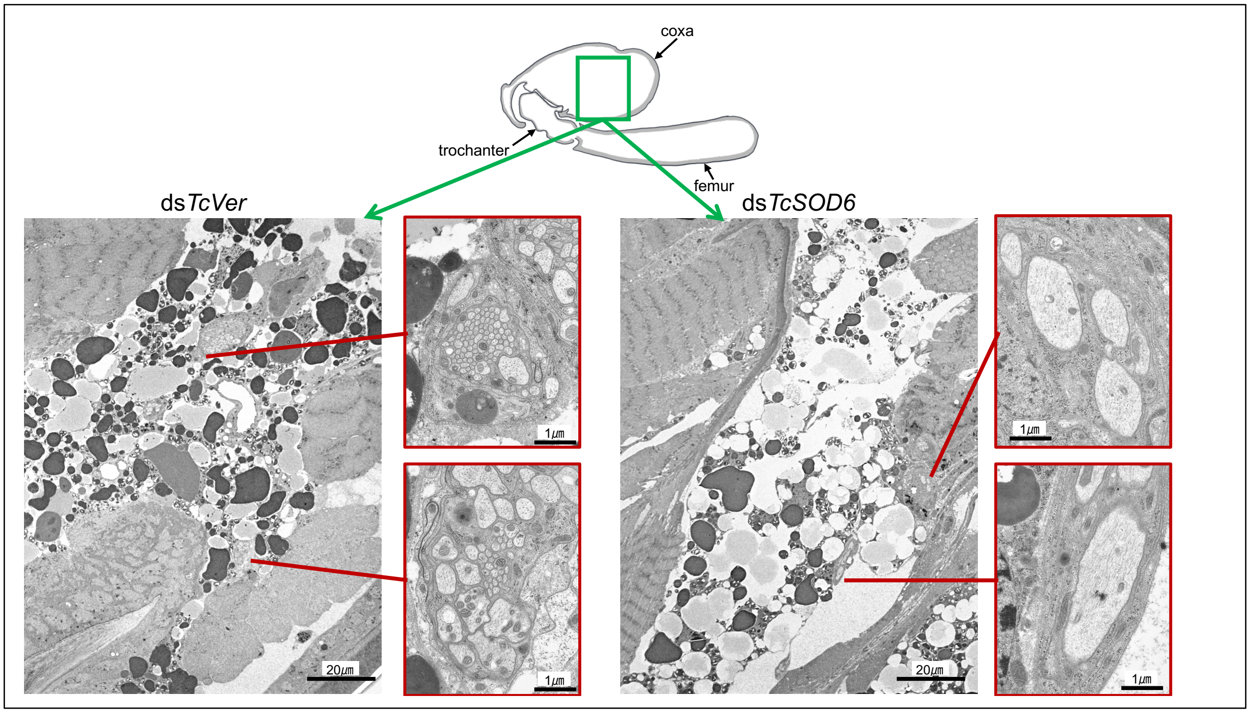

Supplementary Figure 5. Ultrastructural images of coxa in *TcVer*-and *TcSOD6*-knockdown hind legs in biological replicant 2. Representative transmission electron microscopy images obtained from day 6 pupae in the *TcVer*- and *TcSOD6*-knockdown groups. The positions of *T. castaneum* adult coxa is shown in illustrations. The left panel shows a *TcVer*-knockdown pupa. Right panels show a *TcSOD6*-knockdown pupa. The high magnification images are shown by the red boxes. Bar=1 µm or 20 µm.

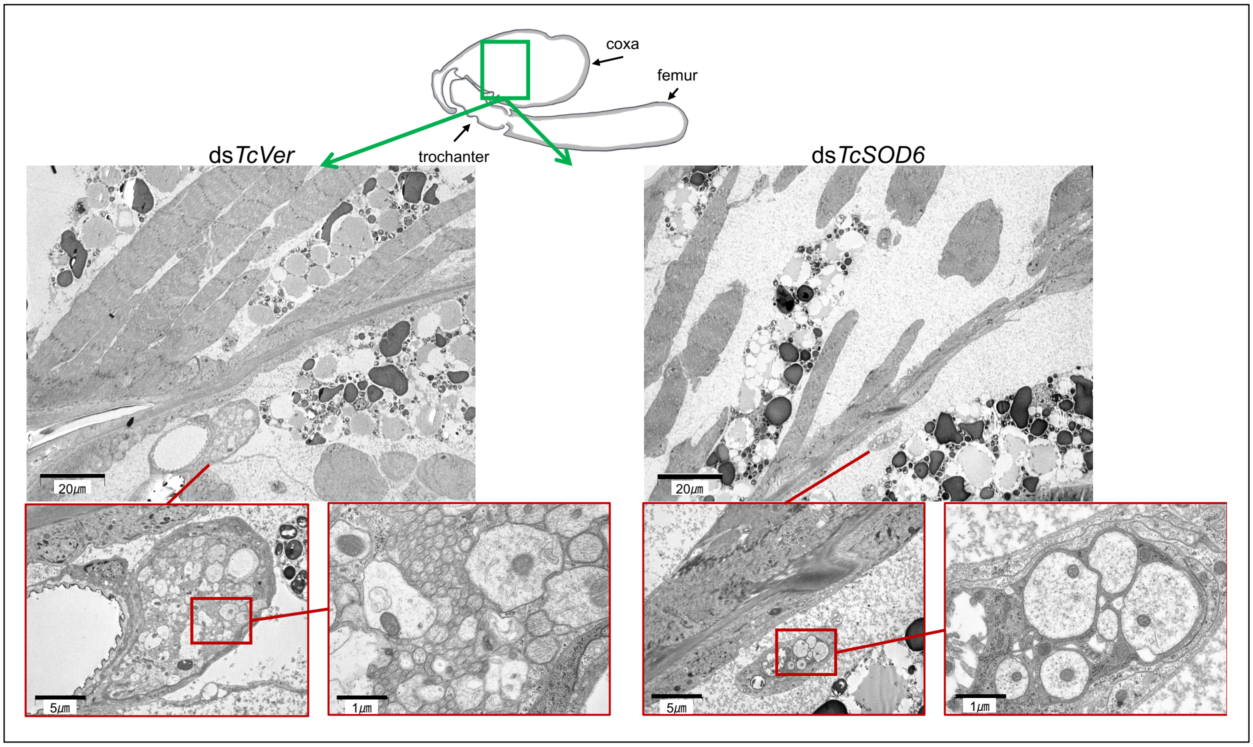

Supplementary Figure 6. Ultrastructural images of coxa in *TcVer*-and *TcSOD6*-knockdown hind legs in biological replicant 3. Representative transmission electron microscopy images obtained from day 6 pupae in the *TcVer*- and *TcSOD6*-knockdown groups. The positions of *T. castaneum* adult legs are shown in illustrations. The left panel shows a *TcVer*-knockdown pupa. Right panels show a *TcSOD6*-knockdown pupa. The high magnification images are shown by the red boxes. Bar=1 µm or 20 µm.

Tables

Table S1. ***SOD*** **sequence IDs obtained from BeetleBase.**

SOD name	Position	Length	Beetle base ID	NCBI ID
TcSOD1	ChLG4:13586289..13586750	462	TC007011	XP_968284
TcSOD2	ChLG8:9478941..9479642	702	TC005780	XP_972440.1
TcSOD3	ChLG9:20121715..20122308	594	TC011676	NP_001164126.1
TcSOD5	ChLG9:20124698..20125624	927	TC011675	XP_972244.1
TcSOD6	ChLG9:18441998..18445842	3845	TC011770	XP_008198302.1
TcCCS	ChLG7:15791708..15792438	731	TC010027	XP_975577.1

Table S2. Gene ID was used the phylogenic tree in this study.

Species	Gene name	Accession ID or Transcriptome IDs
Apis mellifera	AmSOD1	NP_001171498
A.mellifera	AmSOD6	XP_006558396
A.mellifera	AmCCS	XP_006559762
Bombyx mori	BmSOD1	NP_001037084.1
B.mori	BmSOD3	XP_004932190.1
B.mori	BmSOD4	LC229590(*
B.mori	BmSOD5	LC229591(*
B.mori	BmSOD6	LC229592(*
B.mori	BmCCS	LC229593 (*
Drosophila melanogaster	DmSOD1	NP_476735.1
D.melanogaster	DmSOD3	NP_725046
D.melanogaster	DmSOD5	NP_651440.1
D.melanogaster	DmSOD6	NP_733352.3
D.melanogaster	DmCCS	NP_001163108
Danio rerio	DrSOD1	NP_571369.1
D.rerio	DrSOD1	NP_571369.1
D.rerio	DrSOD3a	NP_001092706
D.rerio	DrSOD3b	XP_001332758.1
D.rerio	DrCCS	NP_001191151.2
Homo sapience	HsSOD1	NP_000445
H.sapience	HsSOD3	NP_003093.2
H.sapience	HsCCS	NP_005116
Mus musculus	MmSOD1	NP_035564.1

M.musculus	MmSOD3	NP_035565.1
M.musculus	MmCCS	NP_058588.1
Rattus norvegius	RnSOD1	NP_058746.1
R.norvegius	RnSOD3	NP_037012.1
R.norvegius	RnCCS	NP_445877.1
Tribolium castaneum	TcSOD1	XP_968284
T.castaneum	TcSOD3	NP_001164126.1
T.castaneum	TcSOD5	XP_972244.1
T.castaneum	TcSOD6	LC430326(*
T.castaneum	TcCCS	XP_975577.1
Teleogryllus occipitalis	ToSOD1	jg63502.t1(**
T.occipitalis	ToSOD3	jg43035.t1(**
T.occipitalis	ToSOD3	jg38969.t1(**
T.occipitalis	ToSOD6	jg24026.t1(**
T.occipitalis	ToCCS	jg61478.t1(**
T.occipitalis	ToCCS	jg33379.t1(**
Xenopus tropicalis	XtSOD1	NP_001016252.1
X.tropicalis	XtSOD3	NP_001106630.1
X.tropicalis	XtCCS	NP_001011020
Anopheles gambiae	AgSOD1 isoformA	XP_001230820
A.gambiae	AgSOD1 isoformB	XP_311594
A.gambiae	AgSOD3	XP_314137
A.gambiae	AgCCS	XP_308747.4
A.gambiae	AgSOD6	XP_321476
A.gambiae	AgSOD5	XP_308380

*: DDBJ Gene IDs; **: Transcriptome IDs for *Teleogryllus occipitalis* (cricket) were obtained from the BioProject PRJDB9056.

Table S3. Observation for the leg movement when *TcSOD6* dsRNA was injected at prepupa.

individual	TcSOD6 knockdown			TcVer knockdown		
	T1	T2	T3	T1	T2	T3
1	N	Y	Y	N	N	N
2	N	Y	Y	N	N	N
3	Y	Y	Y	N	N	N
4	N	Y	Y	N	N	N
5	N	Y	Y	N	N	N
6	Y	Y	Y	N	N	N

7	Y	Y	Y	N	N	N
8	N	Y	Y	N	N	N
9	N	Y	Y	N	N	N
10	Y	Y	Y	N	N	N
11	N	Y	Y	N	N	N
12	Y	Y	Y	N	N	N

Observation of "abnormal leg movement" indicates Y, "Not detect" indicates N.

Table S4. Observation for the leg movement when *TcSOD6* dsRNA was injected at day 0 adult.

individual	TcSOD6 knockdown			TcVer knockdown		
	T1	T2	T3	T1	T2	T3
1	N	N	N	N	N	N
2	N	N	N	N	N	N
3	N	N	N	N	N	N
4	N	N	N	N	N	N
5	N	N	N	N	N	N

Observation of "abnormal leg movement" indicates Y, "Not detect" indicates N.

Table S5. TcSOD6 primers used in cDNA cloning

Gene name	Forward	Reverse
TcSOD6	5'-ATGGGACAAATTATCTTCGT-3'	5'-TTACAATAAATAGCTTAAAA-3'

Table S6. TcSODs primers used in quantitative reverse transcription PCR

Gene name	Forward	Reverse
TcRpS6	5'-GGACCCAAAAGAGCATCAAA-3'	5'-CCTCAAGCAACGCTTCTTCT-3'
TcSOD1	5'-GGTGATGGAGTTGCCAAAGT-3'	5'-GATTCCAACAACACCACACG-3'
TcSOD2	5'-CAAGTCCGGGAAATTGAAAA-3'	5'-CTCGAAAATGGCCTTGACAT-3'
TcSOD3	5'-CTTCCACATCCACGAGAAGG-3'	5'-CACCACAGCCCTCCCTATAA-3'
TcSOD5	5'-ACCAAAGCGACCTATACGAA-3'	5'-GCTGCACCCTTGTCTCAAAT-3'
TcSOD6	5'-GGCAAGATTGCAGACAGTGA-3'	5'-AACCAATCCTTGACCACAGC-3'
TcCCS	5'-CCTTATGGGGACTTGGGAGT-3'	5'-CTCTGGGTGGGATTTTCTCA-3'
TcVer	5′-CGTAATGAGTTGCCCACTGAGAC-3′	5′-CGACTCTTCTAAAACGTCGCTGA-3′

Table S7. Primers used for dsRNA synthesis

Target gene	Forward	Reverse

TcSOD6	5’-CACAGCCAAAGTCTGGACTT-3’	5’-GGACTGTATTATAACCTTGG-3’
TcSOD6 with T7	5’-TAATACGACTCACTATAGGCACAGCCAAAGTCTGGACTT-3’	5’-TAATACGACTCACTATAGGGGACTGTATTATAACCTTGG-3’
TcVer	5’-TGATCCTCAAGGTATTGGTG-3’	5’-TGATCAGCGAGTCTATGTCC-3’
TcVer with T7	5’-TAATACGACTCACTATAGGTGATCCTCAAGGTCTTGGTG-3’	5’-TAATACGACTCACTATAGGTGATCAGCGAGTCTATGTCC-3’

Supplementary Video 1. TcSOD6-knockdown insect movement after dsRNA injection in the prepupa.

Supplementary Video 2. TcVer-knockdown insect movement after dsRNA injection in the prepupa.

Supplementary Video 3. TcSOD6-knockdown insect movement following dsRNA injection immediately after adult eclosion.

Supplementary Video 4 Video. TcVer-knockdown insect movement following dsRNA injection immediately after adult eclosion.
